# Acute Psychosis and Mania: An Uncommon Complication of Dengue Fever

**DOI:** 10.7759/cureus.47425

**Published:** 2023-10-21

**Authors:** Zenia Elavia, Soumya Suvra Patra, Sumit Kumar, Pugazhendi Inban, Mohammed Adnaan Yousuf, Ishaan Thassu, Hassan A Chaudhry

**Affiliations:** 1 Neurology, Dr. D. Y. Patil Medical College, Hospital & Research Centre, Pune, IND; 2 Internal Medicine, Calcutta National Medical College, Kolkata, IND; 3 Medicine, Armed Forces Medical College, Pune, IND; 4 General Medicine, Government Medical College, Omandurar, Chennai, IND; 5 Internal Medicine, Kamineni Institute of Medical Sciences, Narketpally, IND; 6 Biological Sciences, Temple University, Philadelphia, USA; 7 Medicine, Medical University of Lublin, Lublin, POL

**Keywords:** enzyme-linked immunosorbent assay (elisa), encephalitis lethargica, affective disorders, schizophrenia, dengue fever

## Abstract

Dengue fever is primarily known as a mosquito-borne viral infection that causes a range of physical symptoms, ranging from myalgia to bleeding tendencies. However, there is growing recognition of its potential to trigger psychiatric manifestations, although such cases remain relatively rare. We report a case of acute dengue fever in a 25-year-old male who developed mania and psychotic symptoms after one week of infection. A comprehensive diagnostic workup, including laboratory tests, including cerebrospinal fluid analysis, and neuroimaging, confirmed the absence of organic causes for his psychiatric symptoms, except for his prior exposure to the dengue virus. The patient was initiated on mood stabilizers and antipsychotic medications, leading to a gradual improvement in his mental health.

## Introduction

Dengue fever, a mosquito-borne infection, can manifest in a variety of symptoms, including high fever, headaches, joint and muscle pain, rash, and bleeding tendencies. Notably, it can also involve neurological symptoms, adding complexity to its clinical presentation [1]. Among cases of dengue fever, neurological manifestations are observed in a range of 0.5% to 5.4% [2,3]. As early as 1845, Esquirol et al. proposed the concept of the infective origins of mental illness. Notably, patients with encephalitis lethargica displayed characteristics reminiscent of schizophrenia and affective disorders [3]. Recently, the interface between infectious diseases and mental health has garnered escalating attention within the medical sphere. Dengue fever, common in tropical areas, is gaining attention for its diverse neurological symptoms; its previously cyclic pattern is now annual [4]. There have been an increasing number of reports in recent years of dengue fever with atypical presentations, particularly those with neurological symptoms [1,4]. The physiological impact of dengue fever is well documented, and the potential role of dengue fever in precipitating psychiatric symptoms is a need for exploration. Despite documented instances of encephalitis linked to dengue fever, the broader spectrum of psychiatric manifestations remains underrepresented in the literature [5]. Here, we report a case of the first episode of mania following serologically confirmed dengue fever and discuss possible treatment and the course of the illness.

## Case presentation

A 25-year-old male was brought to the psychiatry department with a sudden onset of manic behavior and psychotic symptoms following a recent episode of dengue fever. He was previously in his usual state of health until one week ago when he contracted dengue fever during a visit to a tropical region. He presented with classic symptoms of dengue, including high-grade fever, a severe headache, joint pain, and a rash. He did not report bleeding from any site. He was managed conservatively with supportive care in the outpatient setting with paracetamol.

However, approximately five days after three days of his acute dengue symptoms, his family noticed a significant change in his behavior. He displayed extreme hyperactivity, impulsivity, pressured speech, and decreased need for sleep. He also exhibited delusions of grandeur, claiming to have special powers and the ability to communicate with supernatural entities. Labile mood swings and irritability accompanied these psychotic symptoms. He had no prior history of psychiatric illness or hospitalization. There was no known family history of mood disorders or psychosis. He had no known chronic medical conditions and was not taking any medications, recreational drugs, or alcohol. He reported no history of photophobia, seizure, or overt bleeding. He was a software engineer and led an active lifestyle. He is married, and his family reported that there have been no significant stressors or life changes leading up to the onset of his symptoms.

During the comprehensive mental state examination, the patient was hemodynamically stable but exhibited a body temperature of 100°F and had an average build. His mood was highly elevated, accompanied by pressured speech and conspicuous agitation. This pressured speech suggested an urgent and rapid manner of communication, which was congruent with his emotionally elevated state, indicative of a manic episode. Furthermore, the patient displayed disorganized thought processes, reflecting a lack of logical coherence. His thought patterns appeared tangential or characterized by racing thoughts, making it challenging to discern a logical, coherent flow of ideas. Despite these disorganized thought processes, the patient's higher mental functions, including abstract thinking, remained intact, signifying his ability to engage in complex, abstract reasoning and cognitive tasks.

Additionally, the Young Mania Rating Score (YMRS) was recorded at 28, pointing to the severity of the manic episode. The YMRS serves as a standardized tool to assess the intensity of manic symptoms in individuals with mood disorders, and a score of 28 indicates a profound degree of mania. In conjunction with the YMRS, the patient underwent a Mini-Mental State Examination (MMSE), obtaining a score of 28 out of 30, which suggested the absence of significant cognitive impairment.

**Figure 1:**
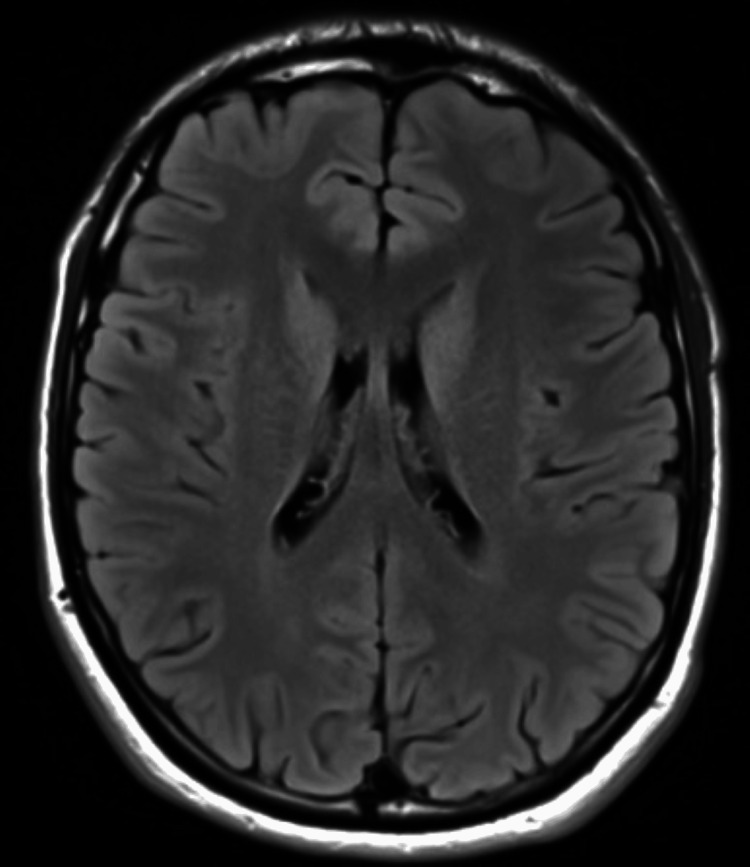
Normal brain MRI demonstrating no abnormalities.

No neurological deficits and signs of meningeal irritation were noted. The rest of the systemic examination was unremarkable. His laboratory evaluations revealed positive immunoglobulin M (IgM) on enzyme-linked immunosorbent assay (ELISA) with a platelet count of 71,000/ul (150,000-350,000). Serum electrolytes, renal and liver function tests, and coagulation profiles were within normal range, and urine analysis was normal. An electrocardiogram revealed sinus rhythm. A brain magnetic resonance imaging (MRI) scan shows no structural abnormalities (Figure 1).

Based on the clinical presentation and laboratory findings, he was diagnosed with a manic episode with psychotic features in the aftermath of dengue fever. He was managed with oral olanzapine 20 mg/day and lorazepam 2 mg/day with close observation and monitoring. Over the course of seven days, a notable improvement was observed, with a 50% reduction in symptoms as assessed by the 11 YMRS score. The benzodiazepine lorazepam was gradually discontinued over two weeks. However, olanzapine was continued for six months. The patient remained asymptomatic after two months of discontinuing the antipsychotic medication. The patient responded well to the treatment given and followed up.

## Discussion

The neurological manifestations associated with dengue infection are progressively becoming more documented [6]. Given its significant impact on public health across many parts of Asia, a comprehensive understanding of the potential neuropsychiatric implications of dengue fever is imperative.

The classic neuropsychiatric signs and symptoms associated with the acute phase of dengue are as follows: headache, seizures, delirium, insomnia, restlessness, irritability, and depression [7]. These can be accompanied by subtle meningism without alteration of consciousness, focal neurological deficits, sensory depression, seizures, and behavioral disorders. In addition, signs of pyramidal and meningeal impairment might be present [8,9]. Among the post-dengue disorders, epilepsy, tremors, amnesia, dementia, manic psychosis, Bell's palsy, laryngeal involvement, lower limb paralysis, palate paralysis, ulnar nerve paralysis, long thoracic nerve paralysis, peroneal nerve paralysis, Reye syndrome, Guillain-Barré syndrome, meningoencephalomyelitis, and mononeuropathies have been reported [10]. 

A recent study reported that among psychiatric presentations, depressive disorders emerged as the most prevalent. Most patients, ranging from 60% to 90% during the acute phase, presented with anxiety and depression symptoms [11]. Syndromal depression was present in 5% to 15% of patients in the convalescent phase. In the acute phase, a substantial number, around 80% to 90%, displayed anxiety symptoms, particularly thanatophobia [11]. Still, these symptoms significantly diminished during the convalescent period, with only 5% experiencing persistent symptoms at the three-month follow-up mark [11]. Another study indicated that during the acute phase, 62% met the criteria for depression, while 59% met the criteria for anxiety. Notably, women displayed more severe depressive symptoms compared to men. Furthermore, the severity of depression, anxiety, and stress negatively correlated with self-efficacy scores during the acute infection period [12]. Our patient with no previous psychiatric history exhibited manic symptoms, including over-talkativeness, heightened religiosity, uncontrollable laughter, and cognitive distortions following dengue fever, implying an infectious origin for his neuropsychiatric presentation as determined by DSM-V criteria [13].

The primary dengue infection management options mainly involve anti-inflammatory agents and corticosteroids. Psychiatric disturbances induced by corticosteroid use are frequently observed and encompass a spectrum of symptoms, such as mania, depression, mixed affective states, psychosis, cognitive impairments, and milder psychiatric issues like irritability, insomnia, anxiety, and mood swings. In children, these effects are often seen as alterations in behavior. However, studies on psychiatric manifestations have not reported management details or specified agents and doses. Antipsychotics, benzodiazepines, or mood stabilizers treat dengue mania [14-16]. Oral antipsychotics at modest dosages worked well for psychotic presentations [17-21]. Intravenous lorazepam (4 mg/day), tapered and terminated over four weeks, helped catatonic patients [22]. Dengue patients with mental comorbidities received clonazepam and low-dose quetiapine without a professional referral. Fewer than 2% of mental morbidity patients were referred for an official examination [23]. The YMRS is a standardized psychiatric assessment tool used to measure the severity of manic symptoms in individuals, and the YMRS is designed to aid clinicians in diagnosing and monitoring manic episodes, particularly in the context of bipolar disorder. It consists of multiple items that assess various aspects of mania, such as elevated mood, energy levels, irritability, and disruptive behavior. The scale's scoring system allows for a quantitative evaluation of manic symptomatology, facilitating more accurate diagnosis and treatment planning for individuals experiencing manic episodes [24]. In our patient, the administration of pharmacotherapy involving olanzapine and lorazepam demonstrated effective management of initial manic symptoms. This treatment yielded promising improvements, as evidenced by a notable reduction in YMRS scores [24].

## Conclusions

Our case highlights the emerging evidence of psychiatric manifestation in dengue fever. Our case provided the intriguing interplay between dengue fever and the subsequent emergence of manic symptoms with psychotic features. Our case emphasizes the need for vigilance in assessing psychiatric symptoms following infectious diseases, even in the absence of a prior psychiatric history. Prompt diagnosis and appropriate management can lead to favorable outcomes in cases where manic and psychotic symptoms emerge in the aftermath of dengue fever.
